# Anatomical Insights into Gamma Knife Radiosurgery: Exploring Efficacy Across Infratentorial and Supratentorial Meningiomas

**DOI:** 10.7759/cureus.83841

**Published:** 2025-05-10

**Authors:** Hoshanc Sdeeq Rashid, Ali Hassan Baker, Salar Tahsin Ismail

**Affiliations:** 1 Department of Neurological Surgery, Erbil Teaching Hospital, Erbil, IRQ; 2 Department of Neurological Surgery, Hawler Medical University, ِErbil, IRQ; 3 Department of Neurological Surgery, Hawler Medical University, Erbil, IRQ

**Keywords:** gamma knife radiosurgery, infratentorial, meningioma, supratentorial, tumor control rates

## Abstract

Gamma Knife radiosurgery (GKRS) is a well-established, minimally invasive treatment modality for meningiomas, offering high rates of tumor control and symptom relief. However, clinical outcomes are influenced by tumor anatomical location, necessitating a comprehensive evaluation of its effectiveness across infratentorial and supratentorial regions.

A systematic review was conducted by following the Preferred Reporting Items for Systematic Reviews and Meta-Analyses (PRISMA) guidelines, including clinical trials and observational studies evaluating GKRS outcomes in meningiomas. Studies were included if they explicitly stratified results by anatomical location and reported clinical outcomes such as tumor control, progression-free survival (PFS), symptom relief, and complications. The ROBINS-I (Risk of Bias in Non-randomized Studies - of Interventions) tool was used to assess the risk of bias. Data were synthesized qualitatively, and outcomes were compared between anatomical locations. Eighteen studies involving 2,257 patients were included. Overall five-year tumor control rates were high for both infratentorial (93.2%) and supratentorial (91.4%) meningiomas. Infratentorial tumors showed slightly better control rates but higher complication rates, including peritumoral edema and cranial nerve deficits due to proximity to critical structures. Supratentorial meningiomas demonstrated favorable long-term outcomes with fewer complications.

Infratentorial tumors achieve excellent control but present higher risks of complications, partly due to their proximity to critical structures and the need for precise dosimetry in typically smaller tumors. In contrast, supratentorial tumors, often larger and more accessible, benefit from fewer adverse events and comparable long-term outcomes. Tumor size, along with anatomical location, plays a key role in radiosurgical planning and complication risk. Future research should focus on prospective studies to refine treatment protocols and enhance outcomes in atypical and anaplastic meningiomas.

## Introduction and background

Meningiomas, accounting for approximately one-third of all primary central nervous system (CNS) tumors, are the most prevalent intracranial tumors, often arising from arachnoid cap cells of the meninges. While most are benign and classified as WHO Grade I, atypical (Grade II) and anaplastic (Grade III) meningiomas exhibit more aggressive behavior, higher recurrence rates, and pose significant therapeutic challenges [1]. Advances in imaging and surgical techniques, coupled with non-invasive modalities like Gamma Knife radiosurgery (GKRS), have revolutionized meningioma management. However, their anatomical location, histopathological grade, and patient-specific factors influence treatment outcomes and prognosis [2].

Anatomical localization also plays a crucial role because the distribution within the CNS is heterogeneous. The most frequent locations are the supratentorial ones, such as the parasagittal, falcine, and convexity positions, with infratentorial ones occurring within the posterior fossa [3]. The distribution determines surgical accessibility and feasibility for radiosurgery as well as therapeutic outcomes. Furthermore, non-skull base meningiomas have a higher likelihood than their skull base counterparts to be malignant or atypical and therefore need aggressive therapeutic approaches [3].

GKRS has emerged as a crucial treatment option for meningiomas, providing high rates of tumor control with minimal invasiveness. Its effectiveness is, nonetheless, dependent upon tumor grade, tumor size, and anatomical complexity, most notably in complicated areas like the base of the skull. Nevertheless, the relationship between the anatomy site, tumor behavior, and radiosurgical factors remains a subject of research [1-3]. The current study aimed to provide a comprehensive evaluation of the results after GKRS for meningiomas, categorized based on their site of anatomy.

## Review

Methods

**Figure 1:**
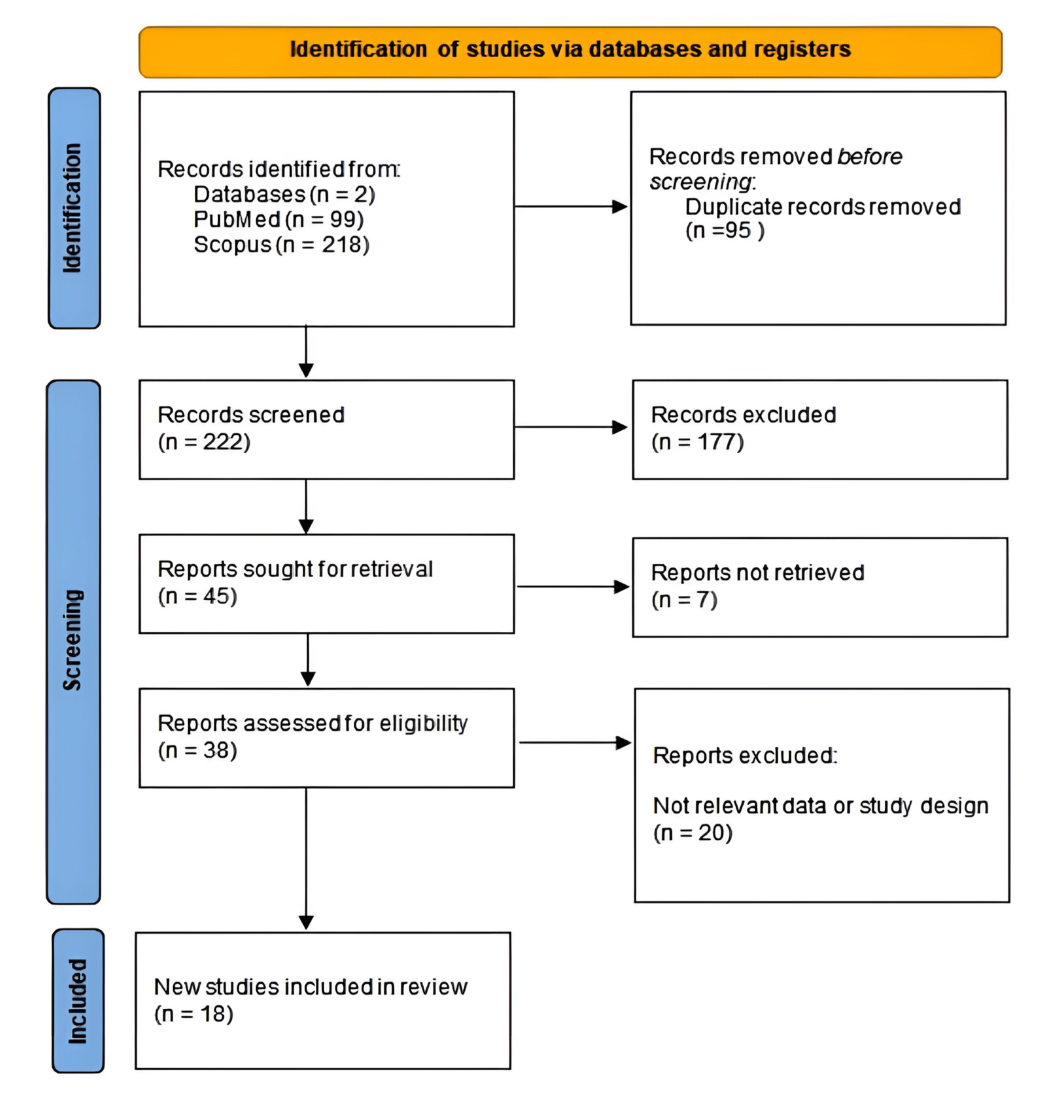
PRISMA flowchart depicting the inclusion of studies PRISMA: Preferred Reporting Items for Systematic Reviews and Meta-Analyses

This systematic review was conducted following the guidelines of the Preferred Reporting Items for Systematic Reviews and Meta-Analyses (PRISMA) to ensure comprehensive and systematic reporting of the evidence synthesis process. Firstly, 317 records were retrieved from two databases: PubMed (n=99) and Scopus (n=218). After removing 95 duplicate records, 222 unique articles were screened based on titles and abstracts. Of these, 177 were excluded due to irrelevance. The full texts of the remaining 45 articles were retrieved, and 38 were assessed for eligibility. After excluding 20 articles due to inappropriate study design or lack of stratification by anatomical location, 18 studies met all inclusion criteria and were ultimately included in the review (Figure 1) [4].

Search Strategy

A comprehensive search was performed using the PubMed and Scopus databases to identify relevant original research articles. The search terms were ( "Gamma knife" OR "Radiosurgery" ) AND ( "Meningioma" ) AND ( "Location" ) AND ( "Outcome" ). The search included all eligible studies published up to November 2024, with no restrictions on the starting year.

Screening and Selection

The Rayyan application was used to manage the multi-step screening process. It allowed two independent reviewers to blind screen titles and abstracts, label studies as "included", "excluded", or "maybe", and resolve conflicts through consensus or third-party adjudication. The tool’s tagging, filtering, and blinding functions enhanced accuracy and transparency in the selection process. The review included clinical trials and observational studies evaluating the outcomes of GKRS for meningiomas and explicitly stratified results by anatomical location. Studies were eligible if they reported clinical outcomes, such as tumor control, symptom relief, recurrence, or survival rates. Reviews, meta-analyses, editorials, or case reports, as well as studies lacking explicit stratification by anatomical location or detailed outcome data, were excluded. Articles unrelated to GKRS were also excluded from this review.

Titles and abstracts were screened by two independent reviewers. Full texts of potentially relevant studies were assessed against the inclusion and exclusion criteria. Discrepancies during screening or selection were resolved by consensus or consultation with a third reviewer.

Risk of Bias Assessment

The risk of bias in included studies was assessed using the ROBINS-I (Risk of Bias in Non-randomized Studies - of Interventions) tool [5]. Each study was evaluated across seven domains, including confounding, selection of participants, classification of interventions, and outcome measurement. Risk levels were categorized as low, moderate, serious, or critical. Discrepancies were resolved through discussions.

Data Extraction

Data extraction was performed independently by two reviewers using a standardized data extraction form. Key variables included study characteristics (design, sample size, follow-up duration), anatomical location, GKRS parameters, clinical outcomes and complication rates, and adverse events.

Data Synthesis

Qualitative synthesis was conducted to summarize the findings, with particular focus on differences in outcomes between infratentorial and supratentorial meningiomas. Data were presented in descriptive tables and grouped by anatomical location. Quantitative pooling of results was not performed due to heterogeneity in study populations, radiosurgical techniques, and outcome measures.

Results

This systematic review included 18 studies comprising 2,257 patients treated with GKRS for meningiomas (Table 1) [6-23].

**Table 1:** Summary of clinical outcomes of Gamma Knife radiosurgery for meningiomas stratified by location and study parameters ARE: adverse radiation effects; BED: biologically effective dose; CN: cranial nerve; CPA: cerebellopontine angle; D98%: dose covering 98% of the target volume; Dose prescription: (e.g., 50% isodose line); GKR: Gamma Knife radiosurgery; GKS: Gamma Knife surgery; IQR: interquartile range; isodose: percentage of the maximum radiation dose received by a specific area; LC: local control; Marginal dose: radiation dose at the periphery of the target tumor; Max dose: maximum radiation dose delivered; N: Number of patients included in the study; nV14Gy: volume receiving 14 Gy dose; PFS: progression-free survival; PTE: peritumoral edema; peripheral dose: dose delivered to the edges of the tumor; recurrence rate: percentage of cases where the tumor reappears post-treatment; SRS: stereotactic radiosurgery; tumor control: ability to prevent tumor progression or recurrence; WHO: World Health Organization

Study ID	Author(s), year	Design	N	Location	Tumor grade/size	GKR parameters	Follow-up	Symptom relief	Complications	Recurrence	Survival
1	Iwai et al., 1999 [6]	Retrospective study	24	Petroclival (11), cavernous sinus (9), cerebellopontine angle (4)	Median volume: 10.9 cm³ (1.2–28.6 cm³); staged treatment for large tumors	Peripheral dose: 8–15 Gy (median 10.6 Gy); single and staged treatments	Median 17.1 months (6–36 months)	46% improved clinically; improvement in trigeminal, oculomotor, and abducens nerve deficits	4% delayed transient radiation injury; 1 patient worsened preexisting cranial nerve deficit	No recurrence; 46% of tumors decreased in size, 54% stable	100% tumor control; effective alternative for surgical removal
2	Nicolato et al., 2002 [7]	Retrospective study	122	Cavernous sinus	Mean volume: 8.3 cm³ (1–20 cm³); Grade I only	Dose: 11–22.5 Gy (mean 14.6 Gy); isodose: 30–65%	Median 48.9 months (12–99 months)	Neurological improvement in 78.5% (primary GKR) and 60.5% (adjuvant GKR); 97% stable/improved overall	4% transient morbidity (edema); 1% permanent morbidity (intracranial hypertension)	Tumor control rate 97.5%; PFS at 5 years: 96.5%	96.5% actuarial survival rate at 5 years
3	Haselsberger et al., 2009 [8]	Retrospective study	20	Critical locations (e.g., cavernous sinus, petroclival)	Median volume: 33.3 cm³ (13.6–79.8 cm³)	Marginal dose: 12 Gy (median); staged treatments	Median 7.5 years	Improvement in 45%; 55% remained stable	10% transient edema resolved with steroids; no permanent neurological deficit; low morbidity	90% tumor control; stable in 65%, regression in 25%	Long-term tumor control; safe for large critical tumors
4	Kondziolka et al., 2009 [9]	Retrospective study	125	Convexity regions (frontal, parietal, temporal, occipital)	Mean tumor volume: 7.6 ml (range 0.4–56.5 ml); WHO Grades I, II, III included	Marginal dose: mean 14.2 Gy; max dose: 28.2 Gy; isodose: 50%	Median 31 months	92% tumor control overall; for Grade I, 95.3% control rate; improved in most benign cases	9.6% morbidity, 4.8% permanent; 5% symptomatic edema; transient headaches, seizures	Tumor control: 97% for Grade I; 50% for Grade II; 0% for Grade III	Effective long-term for Grade I tumors; outcomes decline in higher-grade tumors
5	Flannery et al., 2010 [10]	Retrospective study	168	Petroclival region, middle cranial fossa, cavernous sinus, prepontine space	Median tumor volume: 6.1 cm³ (range 0.3–32.5 cm³); WHO Grade I–III	Marginal dose: 13 Gy (range 9–18 Gy); prescribed to 50% isodose line	Median 72 months	26% neurological improvement, 58% stable, 15% worsened; 91% PFS at 5 years, 86% at 10 years	8% adverse radiation effects; 2% cranial nerve dysfunction; symptomatic hydrocephalus in 4%; low morbidity overall	10% tumor volume increase requiring additional management strategies	91% tumor control for benign cases; poor prognosis for higher-grade tumors
6	Ganz et al., 2010 [11]	Retrospective study	67	Anterior visual pathway (cavernous sinus, tuberculum sellae, sphenoidal ridge)	Median volume: 8.9 cm³ (varied); Grade I	Marginal dose: 12 Gy (9–12 Gy range); isodose 50%; sub-optimal dosimetry used	Median 47 months (25–79 months)	39% improved vision, including diplopia; no visual deterioration reported	3 cases with dose exceeding 9 mm³ of 8–10 Gy tolerance; transient mild edema	100% growth-free survival at 2 years; shrinkage in 46% of cases; no regrowth reported	Safe preservation of visual function; high tumor control with adaptive dosimetry
7	Starke et al., 2011 [12]	Retrospective study	152	Posterior fossa (CPA, tentorium, clival, petroclival)	Median tumor volume: 5.7 cm³ (range 0.3–33 cm³); WHO Grade I only	Marginal dose: 14 Gy (range 8–25 Gy); max dose: 34 Gy (12–60 Gy); isodose 43% (range 25–60%)	Median 7 years (range 2–16 years)	91% with stable or improved symptoms, 36% no tumor change, 51% decreased volume	5.3% edema, 2% hydrocephalus requiring shunt, 9% overall symptom worsening	78% PFS at 10 years	Long-term tumor control with low morbidity, suitable for high-risk surgical candidates
8	Ding et al., 2013 [13]	Retrospective study	65	Parasagittal and parafalcine regions	Median tumor volume: 3 cm³ (range 0.1–15.4 cm³); WHO Grade I	Median marginal dose: 15 Gy (range 10–20 Gy); isodose 40% (30–55%)	Median 75.6 months (6.1–199.5 months)	91% had stable or improved Karnofsky Performance Status; tumor volume was stable or reduced in 85% of cases	8.2% symptomatic postradiosurgery edema (1 permanent); 14.3% postradiosurgery seizures	Tumor control 85% at 3 years, 70% at 5 years; better outcomes in younger patients and no prior resection cases	Safe long-term management for WHO Grade I tumors with moderate complications
9	Starke et al., 2014 [14]	Multicenter retrospective study	254	Petroclival meningiomas	Mean tumor volume: 7.8 cm³ (range 0.17–36.1 cm³); WHO Grade I	Median margin dose: 13.4 Gy (range 9–40 Gy); max dose: 27.6 Gy (range 18–80 Gy); isodose: 48.9%	Mean 71 months (range 6–252 months)	39% tumor shrinkage, 52% stable size; 93.6% of patients showed stable/improved neurological function	2.8% hydrocephalus requiring shunt, 6.4% experienced new/worsened neurological deficits	PFS: 93% at 5 years, 84% at 10 years	High tumor control rate with minimal complications, suitable for complex locations
10	Sheehan et al., 2015 [15]	Multicenter retrospective study	675	Posterior fossa (clival, petroclival, tentorial, cerebellopontine angle)	Mean tumor volume: 6.5 cm³ (range 0.15–41.8 cm³); WHO Grade I	Marginal dose: 13.6 Gy (range 8–40 Gy); max dose: 28.6 Gy (mean); isodose 48.5%	Median 60 months (range 6–252 months)	Tumor control in 91.2%; 92.3% stable/improved neurological function	8.8% tumor progression; 7.7% experienced new or worsening neurological deficits; 1.7% hydrocephalus requiring shunt	95%, 92%, and 81% PFS at 3, 5, and 10 years, respectively	High tumor control and neurological preservation; low incidence of severe complications
11	D'Amico et al., 2017 [16]	Retrospective study	76	Cerebellopontine angle (CPA) meningiomas	Median tumor size: 3.0 cm (range 0.8–6.5 cm); WHO Grade I and II	Median marginal dose for GKR: 14 Gy (range 12–16 Gy)	Median 46 months (range 2–259 months)	89.5% of patients had stable or improved facial nerve function post-treatment	10.5% experienced worsened facial nerve function; hydrocephalus requiring shunt occurred in 5.9% of cases	15.8% recurrence; no progression in patients treated with GKR as primary therapy	Excellent disease control with GKR for small lesions; combined approach is safe for larger tumors
12	Cohen-Inbar et al., 2018 [17]	Retrospective study	189	Parasellar region (cavernous sinus, petroclival, suprasellar)	Median tumor volume: 5.6 cm³ (range: 0.2–54.8 cm³); WHO Grade I	Median marginal dose: 14 Gy (range 5–35 Gy); max dose: 32 Gy	Median 71 months (range 6–298 months)	91.5% achieved tumor control (48.1% regression, 43.4% stability); 39.2% neurological improvement	2.64% SRS-related complications; 9.3% new CN deficits due to SRS, primarily trigeminal and optic	8.5% progression (4.2% infield, 4.2% out-of-field recurrences); long-term control enhanced with doses ≥16 Gy	Excellent tumor control; reliable for high-risk regions, with minimal complications
13	Hasegawa et al., 2018 [18]	Retrospective study	67	Skull base (69%), parasagittal (31%)	Median tumor volume: 4.9 cm³ (range 0.7–22.9 cm³); WHO Grade I	Median marginal dose: 16 Gy (range 12–18 Gy)	Median 52 months (range 7–195 months)	91% stable/improved neurological function; mild/marginal tumor control is effective for older patients	15% peritumoral edema; 13% adverse effects (mild/moderate); higher doses increased risk of complications	Actuarial control rates: 92% at 3 years, 86% at 5 years, and 72% at 10 years	Effective for elderly patients with low morbidity and high tumor control rates
14	Mehta et al., 2018 [19]	Multicenter retrospective study	57	Foramen magnum meningiomas (anterior, anterolateral, posterior)	Median tumor volume: 2.9 cm³ (range 0.4–17 cm³); WHO Grade I	Median margin dose: 12.5 Gy (range 10–16 Gy); max dose: 25 Gy	Median 53 months (range 6–196 months)	52% symptom improvement; most improvement in headaches (85% of cases)	2% adverse radiation effects; rare cases of transient numbness and hearing loss	7% tumor progression; 93% tumor control at 5 and 10 years	Excellent tumor control; safe and effective for small-to-moderate lesions in critical areas
15	Dedeclusova et al., 2022 [20]	Retrospective study	46	Posterior cranial fossa (petrous, petroclival, CPA, foramen magnum)	Median tumor volume: 2.21 cm³ (range 0.3–8.9 cm³); WHO Grade I	Median marginal dose: 12 Gy (range 12–14 Gy); mean BED 63.6 Gy	Median 45.5 months (range 6–108 months)	93.6% tumor control; 25.6% neurological improvement, 71.8% stable; highest clinical improvement with BED of 56–61 Gy	2 cases of asymptomatic peritumoral edema; no severe radiation-induced effects	6.4% tumor progression; actuarial 5-year PFS: 94%	Effective for PCFM with excellent tumor control and minimal complications
16	Islim et al., 2022 [21]	Retrospective matched cohort study	84	Frontobasal (olfactory groove, tuberculum sellae, planum sphenoidale)	Median volume: 3.0 cm³ (IQR 1.0–7.0 cm³); WHO Grade I	Median marginal dose: 12 Gy; max dose: 25 Gy	Median 42 months (range 24–72 months)	Tumor control: 100% with SRS; active monitoring showed 52% tumor progression	6% adverse symptoms in SRS cohort (headache, seizures, mild edema), all resolved; none in surveillance	No progression with SRS; 12% required intervention after surveillance	SRS achieves superior tumor control; active surveillance is appropriate with low intervention needs
17	Lee et al., 2023 [22]	Retrospective study	55	Non-skull base, non-perioptic, supratentorial	Median tumor volume: 4.2 cm³ (range 0.2–31.7 cm³); WHO Grade I	Marginal dose: 14.3 Gy (range 9–20 Gy); single-session GKS	Median 159 months (range 120–226 months)	73.5% PFS at 15 years; LC improved with D98% > 11 Gy	23.5% adverse effects, with 10.3% symptomatic AREs; PTE predictors include nV14Gy > 0.66 cm³	23.5% progression overall; LC rates: 88.2% (5 years), 80.9% (10 years)	Effective for long-term LC with significant dosimetric and tumor factors impacting outcomes
18	Umekawa et al., 2023 [23]	Retrospective study	11	Intraventricular (trigone, body, third ventricle)	Median volume: 4.9 cm³ (range 1.2–9.8 cm³); WHO Grade I	Marginal dose: 14 Gy (post-2010) and 16 Gy (pre-2010)	Median 52 months (range 3–353 months)	58% tumor shrinkage, 42% stable; 100% tumor control	33% transient perifocal edema (8% symptomatic); all resolved without surgery	No tumor progression; cumulative 5- and 10-year progression-free rates: 100%	Excellent tumor control with minimal complications; suitable for deep-seated tumors

The data were stratified based on the anatomical location of the tumors, focusing on infratentorial and supratentorial meningiomas. Outcomes of interest included tumor control, progression-free survival (PFS), and the incidence of complications, with emphasis on location-specific differences. The quality assessment of the included studies revealed a moderate risk of bias across most studies. Key concerns included variability in the reporting of clinical outcomes and potential confounding factors due to differences in tumor characteristics and treatment protocols. However, all studies demonstrated robust methodological designs, ensuring reliable data for evaluating Gamma Knife Radiosurgery outcomes in meningiomas (Table 2) [6-23].

**Table 2:** ROBINS-I assessment of the included studies ROBINS-I: Risk of Bias in Non-randomized Studies - of Interventions

Study ID	Author(s), year	Confounding	Selection of patients	Classification of interventions	Deviations from intended interventions	Missing data	Measurement of outcomes	Selection of reported results
1	Iwai et al., 1999 [6]	Moderate	Low	Low	Low	Low	Moderate	Moderate
2	Nicolato et al., 2002 [7]	Low	Moderate	Low	Low	Low	Moderate	Moderate
3	Haselsberger et al., 2009 [8]	Moderate	Moderate	Low	Low	Moderate	Moderate	Moderate
4	Kondziolka et al., 2009 [9]	Moderate	Moderate	Low	Low	Moderate	Moderate	Moderate
5	Flannery et al., 2010 [10]	Moderate	Moderate	Low	Low	Moderate	Moderate	Moderate
6	Ganz et al., 2010 [11]	Moderate	Low	Low	Low	Moderate	Moderate	Moderate
7	Starke et al., 2011 [12]	Moderate	Moderate	Low	Low	Moderate	Moderate	Moderate
8	Ding et al., 2013 [13]	Moderate	Low	Low	Low	Moderate	Moderate	Moderate
9	Starke et al., 2014 [14]	Moderate	Moderate	Low	Low	Moderate	Moderate	Moderate
10	Sheehan et al., 2015 [15]	Moderate	Moderate	Low	Low	Moderate	Moderate	Low
11	D’Amico et al., 2017 [16]	Moderate	Moderate	Low	Low	Low	Moderate	Moderate
12	Cohen-Inbar et al., 2018 [17]	Moderate	Moderate	Low	Low	Moderate	Moderate	Moderate
13	Hasegawa et al., 2018 [18]	Moderate	Moderate	Low	Low	Moderate	Moderate	Moderate
14	Mehta et al., 2018 [19]	Moderate	Low	Low	Low	Moderate	Moderate	Moderate
15	Dedeclusova et al., 2022 [20]	Moderate	Moderate	Low	Low	Moderate	Moderate	Moderate
16	Islim et al., 2022 [21]	Moderate	Moderate	Low	Low	Moderate	Moderate	Moderate
17	Lee et al., 2023 [22]	Moderate	Low	Low	Low	Low	Moderate	Moderate
18	Umekawa et al., 2023 [23]	Moderate	Moderate	Low	Low	Moderate	Moderate	Low

Tumor Control at Five Years

The overall tumor control at five years was high across both infratentorial and supratentorial meningiomas. Infratentorial meningiomas demonstrated a cumulative tumor control rate of 93.2%, while supratentorial meningiomas had a slightly lower rate of 91.4% (Table 3) [6-23].

**Table 3:** Tumor control rates for infratentorial and supratentorial meningiomas treated with Gamma Knife radiosurgery

Author(s), year	Infratentorial	Tumour control at 5 years, %	Supratentorial	Tumour control at 5 years, %
Iwai et al., 1999 [6]	15	100	9	100
Nicolato et al., 2002 [7]	0	0	122	96.5
Haselsberger et al., 2009 [8]	5	90	15	90
Kondziolka et al., 2009 [9]	0	0	125	92
Flannery et al., 2010 [10]	168	90	0	0
Ganz et al., 2010 [11]	0	0	67	100
Starke et al., 2011 [12]	152	93.4	0	0
Ding et al., 2013 [13]	0	0	65	70
Starke et al., 2014 [14]	254	93	0	0
Sheehan et al., 2015 [15]	675	92	0	0
D'Amico et al., 2017 [16]	67	100	0	0
Cohen-Inbar et al., 2018 [17]	0	0	189	95.7
Hasegawa et al., 2018 [18]	26	88.46	41	80.49
Mehta et al., 2018 [19]	57	92	0	0
Dedeclusova et al., 2022 [20]	42	92.9	0	0
Islim et al., 2022 [21]	0	0	84	100
Lee et al., 2023 [22]	0	0	55	80.9
Umekawa et al., 2023 [23]	0	0	11	100
Total No of patients	1461		783	

Infratentorial meningiomas, located predominantly in the posterior cranial fossa (e.g., cerebellopontine angle, petroclival, and foramen magnum regions), posed significant challenges due to their proximity to critical neurovascular structures. However, despite these challenges, tumor control rates remained robust. Starke et al. [12] and Starke et al. [14] reported five-year tumor control rates of 93.4% and 93%, respectively, highlighting the consistency of GKRS effectiveness in this region. Dedeclusova et al. [20] reported a tumor PFS rate of 94% at five years, further supporting the efficacy of GKRS for infratentorial meningiomas.

Supratentorial meningiomas within the convexity, falx, parasagittal, and frontobasal sites were associated with somewhat lesser control rates for the tumor, but had overall fewer complications. Cohen-Inbar et al. [17] had a 95.7% control for the tumor after five years, with a high percentage of patients with stable or reduced tumor size. Lee et al. [22] offered 88.2% and 80.9% control for the longer term for the tumor at the five-year and 10-year timepoints, respectively, with durable results for supratentorial meningiomas. Of note, Islim et al. [21] offered 100% control for frontobasal meningiomas with GKRS, highlighting the effectiveness of radiosurgery for treatable anatomical sites.

Progression-Free Survival

PFS was a critical metric reported in several studies. Infratentorial meningiomas demonstrated slightly higher rates of PFS compared to supratentorial cases. Sheehan et al. [15] reported a 10-year PFS rate of 95% for infratentorial tumors, compared to 81% for supratentorial tumors. For supratentorial cases, progression was observed in 23.5% of patients in Lee et al. [22], with better outcomes in patients receiving higher marginal doses (>14 Gy).

Complications

Due to their anatomical complexity, infratentorial meningiomas were associated with a higher incidence of complications, particularly peritumoral edema (PTE) and cranial nerve deficits. Dedeclusova et al. [20] reported PTE in 4.8% of cases, all resolving with conservative management. Cranial nerve deficits, often transient, were reported in 10.5% of cases in D'Amico et al. [16], particularly for cerebellopontine angle tumors.

Complications were rare and were most significantly confined to radiation-induced edema (RIE) in supratentorial meningiomas. Cohen-Inbar et al. [17] reported a 2.64% incidence of RIE, all of which were transient and were managed with the administration of steroids. Symptomatic edema was rare and was identified by Lee et al. [22] with a prevalence of 1.8%, which indicates the favorable safety profile of GKRS for supratentorial locations.

Anatomical and Clinical Considerations

The anatomical complexity of infratentorial tumors influenced treatment planning and outcomes. Posterior cranial fossa tumors often require more precise dosimetric approaches due to the proximity of critical structures such as the brainstem and cranial nerves. Despite these challenges, infratentorial tumors showed slightly better tumor control rates at five years compared to supratentorial tumors. This may reflect the inherent biological behavior of infratentorial meningiomas, which are typically smaller at diagnosis and slower growing.

By contrast, supratentorial lesions, including convexity and parasagittal lesions, were benefited by enhanced accessibility and the capability for the use of wider safety margins with radiosurgery. The lesions were associated with fewer complications and comparable long-term outcomes, with GKRS as a highly valuable therapeutic modality for these locations.

Discussion

Meningiomas have shaped the path of neurosurgery for centuries. The first written documentation was provided by Felix Plater in 1664 and the first successful resection was performed by Zanobi Pecchioli in 1835 [24]. Their 20th century treatment was revolutionized with the introduction by Harvey Cushing who invented the term "meningioma" [24]. The review shows the very high efficacy with which GKRS treats the meningiomas with tumor control >90% after five years for infratentorial and supratentorial sites.

Infratentorial meningiomas achieved slightly better five-year tumor control rates (93.2%) compared to supratentorial meningiomas (91.4%). This difference may be attributed to the biological behavior of infratentorial tumors, which are often diagnosed earlier due to symptom presentation and tend to have slower growth rates. Studies such as Sheehan et al. [15] and Starke et al. [14] reported high PFS rates of 95% and 93%, respectively, for posterior cranial fossa tumors, underscoring the durability of GKRS in these complex regions. In contrast, supratentorial meningiomas with somewhat lower tumor control rates benefited from lower complications and better long-term stability. For instance, Islim et al. [21] and Cohen-Inbar et al. [17] provided excellent tumor control rates (100% and 95.7%, respectively) for frontobasal and parasellar meningiomas, most likely due to the ease with which these locations are amenable to maximal dose delivery.

Complication frequencies were also significantly different according to the site of the tumor. Those infratentorial lesions near critical structures such as the brainstem and the cranial nerves were associated with a higher frequency of complications. For example, Dedeclusova et al. [20] reported 4.8% PTE in posterior fossa cases, all of which were resolved with conservative treatment. On the other hand, supratentorial lesions were associated with fewer complications. Lee et al. [22] reported only 1.8% RIE with symptoms. This reflects the relative safety of GKRS for the treatment of tumors within the more accessible supratentorial compartments.

Anatomical localization of the meningiomas presents a special challenge for radiosurgical treatment. Lesions infratentorial within the posterior fossa require precise dose delivery. Notwithstanding such a challenge, Mehta et al. [19] and Starke et al. [12] have demonstrated excellent control of the tumor (92% and 93.4%, respectively), establishing the role of GKRS as a safe and effective modality for the treatment of such high-risk localizations. Supratentorial tumors, particularly convexity and parasagittal ones, have better anatomical accessibility with the option for larger safety margins during radiosurgery. This likely explains the lower complications occurring in these localizations. Furthermore, the symptoms of the tumors in these localizations are generally late to manifest, providing sufficient time for elaborate radiosurgical planning. The effectiveness of GKRS in the supratentorial localizations is also vouched for by studies such as Ding et al. [13] and Cohen-Inbar et al. [17], which demonstrated high control of the tumor with minimal side effects.

Based upon Naik et al. [25], GKRS demonstrates better control of the tumor for WHO grade I meningiomas with 96.3% control at five years. The outcomes are poorer for higher grades, with 62.5% control at five years for grade II and 10% for grade III. As per Fu et al. [26], GKRS demonstrates high efficacy for asymptomatic meningiomas with 98.3% control of the tumor with 97% PFS at five and 10 years. Complications were observed with PTE developing in 25.4% of patients, with 11.9% with symptomatic PTE. Increased tumor size (≥25 mm) and increased maximum dose (≥34 Gy) were independent predictors for PTE.

The findings of this review should be interpreted in the context of its limitations. Most included studies were retrospective, introducing potential biases related to patient selection and data reporting. Additionally, heterogeneity in treatment protocols, such as variations in marginal dose and fractionation schemes, may have influenced outcomes. Future research should focus on prospective studies with standardized protocols to better evaluate the comparative effectiveness of GKRS in infratentorial versus supratentorial meningiomas.

## Conclusions

Infratentorial meningiomas had slightly better tumor control at the end of five years than supratentorial meningiomas due to the differences in biological behavior and presentation. However, infratentorial tumors also had a higher number of complications since the vital neurovascular structures are closer. Supratentorial meningiomas also had equally good long-term outcomes with fewer complications, which reflects the ease with which radiosurgery planning and delivery are done in these locations. We recommend prospective studies with uniform protocols to optimize GKRS results and further establish its place in the treatment of atypical and anaplastic meningiomas.
